# Thank you to our reviewers

**DOI:** 10.1017/cts.2023.24

**Published:** 2023-04-12

We are indebted to the expert referees who have volunteered their time to review submissions for the *Journal of Clinical and Translational Sciences* in 2022. The editors, authors, and readers are immensely grateful for their thoughtfulness and expertise that have served our journal well.

Maryam Mohamed Abdelkarim

Zainab Abedin

Erin Abu-Rish Blakeney

Reuben Adatorwovor

Sergio Aguilar-Gaxiola

Myles Akabas

Matthew Akiyama

Leah Alexander

Ayham Alkhachroum

Karel Allegaert

Jessica Ancker

Peter Anderson

Michael P. Angarone

Mary Ellen Antowiak

Jalayne Arias

Gladys Asiedu

Jared Auclair

Adriana Baez

Laura Balis

Joyce Balls-Berry

Shweta Bansal

Daniel M. Barnett

Josephine Barnett

John Baumann

Sydney Begerowski

L. Michelle Bennett

Lisa Bernard

Laura Mari Beskow

Eric Beyer

Jiang Bian

Sarah Biber

Barbara Bierer

Hugh Black

Helena Blumen

Leslie Boone

Sue Boren

David Boulware

Kathleen T. Brady

G Brannan

Miriam A. Bredella

Sarah K. Brewer

Tabetha A. Brockman

Birgitta Brott

Jen Brown

Jennifer Swanton Brown

Amanda Bunting

John B. Buse

Karen Calhoun

Christopher Cambron

Kendall M. Campbell

Ricky L. Camplain

Adelita Cantu

Olveen Carasquillo

Savanna Carson

Lori Carter-Edwards

Shannon Casey

David Center

Patricia Chalela

Larry Chambers

Alex C. Cheng

Summer Choudhury

James J. Cimino

Robert A. Clark

Nichelle L. Cobb

Barry Coller

Chiquita Collins

Dan Cooper, MD

Linda Cottler

Valerie Cotton

Margaret Crane

Allison Crawford

Marcus Crede

Deidra Crews

Jennifer Croker

John Cullen

Mollie Cummins

Denise Daudelin

Gaurav Dave

Jonathan Davis

Pamela Davis

Willard Dere

Manisha Desai

Mark Dignan

Rowena Dolor

Jianghu Dong

Carrie Dykes

Brenda Eakin

Milton Mickey Eder

Terri L. Edwards

Peter L. Elkin

Vicki L. Ellingrod

Julie T. Elworth

Amanda Emerson

Felicity T. Enders

Ann Margret Ervin

Kolaleh Eskandanian

Estela Estape

Elizabeth Evans

Alecia Fair

David Fakunle

Megan L. Falsetta

Jessica M. Faupel-Badger

Kevin Fiscella

Daniel Ford

Deborah M. Fournier

Mayu Frank

Stephanie Freel

Stephanie A. Freels

Peter Friedmann

Michael Frohman

Ayorkor Gaba

Mohit Ganguly

Armen Yuri Gasparyan

Cheryl Gatto

Mugur Geana

Mariana Gerschenson

Sue Giancola

Kelly Gleason

William Gluck

Keri Godin

Sarah L. Goff

Mary Goldberg

Mitzi Gonzales

Ruth Gotian

Jennifer Grandis

Susan Grinslade

Michael Steven Gutter

Nathaniel Hafer

Perry Halushka

Colin Halverson

Anna Hansen

Laura N. Hanson

Brett Harnett

Kevin Harter

Anuradha Hashemi

Sarah Hawley

Erin Heckler

Elizabeth Heitman

Suzanne Held, Ph.D.

Emily Hennessy

Paul Hernandez

Rachel Hess

Lisa Hightow-Weidman

Fernando Holguin

Mike Holinstat

Cole Hooley

Verónica Hoyo

Xuemei Huang

Matthew F. Hudson

Ana Iltis

Megan Bennett Irby

Rebecca D. Jackson

Laura P. James

Friederike Jayes

Michelle Jenkerson

Rebecca N. Jerome

Carolynn Thomas Jones

Judith Jones

Felichism W. Kabo

Alex Kaizer

Cathleen Kane

Kim Kaphingst

Anand Karnad

Al-Faraaz Kassam

Shanthini Kasturi

Merle Kataoka-Yahiro

Emma Kay

Jacob Kean

Heidi Keeler

Patricia J. Kelly

Thomas H. Kelly

Nick Kenyon

Philip Kern

Anthony Keyes

Akram Khan

Robert Kimberly

Mary Kleinman

Jacqueline Knapke

Andrew Knighton

H. Robert Kolb

Elizabeth Kopras

Rhonda G. Kost

Jack Kues

Polina Kukhareva

Bethany M. Kwan

Marianna LaNoue

Danielle Laraque-Arena

Bernard LaSalle

Robert Lavieri

Harold Lehmann

Leslie A. Lenert

Shu-Yi Liao

Alexandra F. Lightfoot

Christopher Lindsell

Mike Linke

Nicole Llewellyn

On-Yee (Amy) Lo

Jennifer Lorvick

Emily M. Lund

John Luque

Michael Lyons

Wendy Mack

Jane Mahoney

Morgan Maner

Frank Manion

Paul Marantz

Kris M. Markman

Michelle Y. Martin

Kate Marusina

Michael Mashaal, MD

Barbara Massoudi

Jomol Mathew

Tanya Mathew

Colleen A. Mayowski

Jennifer McCaney

Susanna A. McColley

Wayne T. McCormack

Wayne T. McCormack

Pearl McElfish

Richard McGee

Kathleen M. McGroarty

Linda McManus

Demetria McNeal

Emma Anne Meagher

Valentina Medici

Christina Mehta

Laura Meiners

Jareen Meinzen-Derr

Paul Meissner

Frederick J. Meyers

Lloyd Michener

Freneka Minter

Brooke E.E. Montgomery

Sean Mooney

Justin B. Moore

Hiroki Morizono

Elaine H. Morrato

Cynthia Morris

Teng Moua

Timothy F. Murphy

Maureen Murtaugh

Serge Patrick Nana-Sinkam

Donald E. Nease

Eric Nehl

David Nelson

Heike Newman

Hien Nguyen

Henry Stacy Nicholson

Jacinda Nicklas

Nyiramugisha K. Niyibizi

Rocio Norman

Keith C. Norris

Hany Osman

Marisha Palm

Monique Pappadis

Susan R. Passmore

Sapana Patel

Tanha Patel

Cecilia Patino-Sutton

Thomas Pearson

Clara M. Pelfrey

Ryan Peterson

Erika Pike

Harold Pincus

Tiffany Danielle Pineda

David Pleasure

Roy Poblete

Fred Pryor

Jill M. Pulley

Susan Pusek

Kelli Qua

Suhrud Rajguru

Deepa Rastogi

Lee Ann Riesenberg

Laura Riolobos

Betsy Rolland

Morteza Roodgar

Doris Rubio

Elias Samuels

Fatima Ruiz Sancheznieto

Jonathan Schildcrout

Sarah J. Schlesinger

Linda M. Scholl

Denise Scholtens

Craig Schwalbe

Pich Seekaew

Robert Sege

Steven Seidner

Harry Selker

Harsh S. Shah

Muhammad J. A. Shiddiky

Rebecca Shlafer

Evan Sholle

Mina Silberberg

Carsten Claus Skarke

Hope Smiley-McDonald

Justin Dean Smith

Julian Solway

Stephen Sonstein

Christine Sorkness

Ryan Spellecy

Cathie Spino

Milena Stanojlovic

Justin Starren

Scott Steele

Elizabeth Stevens

Kathleen R. Stevens

Sierra Stites

Yulia A. Strekalova

Catherine Striley

Maran Subramain

Laura Jane Sugarwala

Laura Svetkey

Katherine Sward

Laneshia Tague

Faye Taxman

Monica M. Taylor

Gelise Thomas

Luanne Thorndyke

Jennifer Tiffany

Beth B. Tigges

Umit Topalogu

Robert Toto

Robin Tragus

Nicole Tuitt

Laurene Tumiel Berhalter

Jason Umans

Randy Urban

Elizabeth Vasile

Roger D. Vaughan

Alfred Vitale

Boris Volkov

Vanessa V. Volpe

Russ Waitman

Jeffery Walker

Michael J. Ward

Molly Wasko

Dan A. Waxman

Karen Weavers

Gretchen E. White

Adam Wilcox

Shaina Willen

Cathleen Willging

Karen Wilson

Christine Wood

Kevin Wooten

Alysse Wurcel

Yaomin Xu

Paridokht Zarean

Amir Zeki

Hongbin Zhang

Nanhua Zhang

Kristine Zimmermann

Diana M. Zuckerman

